# Prolonged use of letrozole causes morphological changes on gonads in *Galea spixii*

**DOI:** 10.1590/1984-3143-AR2020-0029

**Published:** 2021-07-12

**Authors:** Maria Angelica Machado Arroyo, Paulo Ramos da Silva Santos, Moacir Franco de Oliveira, Antônio Chaves de Assis

**Affiliations:** 1 Departamento de Cirurgia, Faculdade de Medicina Veterinária e Zootecnia, Universidade de São Paulo, São Paulo, SP, Brasil; 2 Departamento de Ciências Animais, Universidade Federal Rural do Semi-Árido, Mossoró, RN, Brasil

**Keywords:** aromatase, estrogens, ovary, rodents, steroidogenesis, testis

## Abstract

Letrozole is used as a therapeutic agent in reproductive disorders caused by high estrogen levels. Letrozole inhibits cytochrome P450 aromatase and reduces estrogen levels. However, the effects of long-term use on reproductive traits are unknown. The aim of this study was to evaluate the prolonged use of letrozole in the gonads of rodents (Spix's yellow-toothed cavy; *Galea spixii*). Forty-eight rodents (24 males and 24 females) were randomly divided into the treated and control groups. Letrozole administration started at 15 days of age and continued weekly until 30, 45, 90, and 120 days of age. The body, testis, and ovary weights were analyzed, as well as the morphological progression of spermatogenesis and folliculogenesis. Macroscopically, body weight gain and gonads weight were increased in the letrozole group. Microscopically, the ovaries of treated females showed stratified epithelium and a cellular disorder of the tunica albuginea. In the testes of treated males, the development of seminiferous tubules was delayed and sperm was absent. The collective findings indicate that the prolonged use of letrozole alters secondary sexual characteristics, and causes weight gain, reproductive changes, and male infertility.

## Introduction

**Figure 1:**
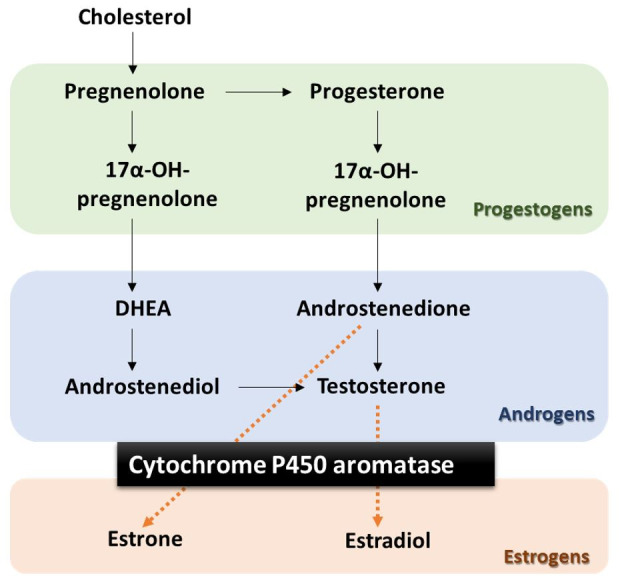
Simplified steroid chain diagram. The highlighted portion is cytochrome P450 aromatase, the enzyme responsible for estrogen biosynthesis from androgens.

Letrozole is a selective P450 aromatase inhibitor that decreases the amount of estrogen produced without changes in other steroidogenic pathways (Lephart, 1996; Bhatnagar, 2007). Aromatase is responsible for the biosynthesis of estrone and estradiol from androgenic hormones, such as androstenedione and testosterone, respectively (Santos et al., 2017b) (Figure 1). Therefore, the use of letrozole allows the evaluation of aromatase activity in vivo, as well as the possible effects of the inhibition of aromatase caused by an imbalance in the ratio of androgens to estrogens (Bhatnagar, 2007).

P450 aromatase is involved in the development of nuclei of sexual dimorphisms and aromatization zones. These zones seem to be related to the control of reproduction and sexual behavior, as described in mammals, amphibians, birds, fish, and reptiles (Corbin et al., 2009; Roselli and Stormshak, 2012). Furthermore, P450 aromatase can also induce follicular and spermatogenesis development, since it exists in the gonads of several species and maintains the physiological proportion between androgens and estrogens, which are directly related to fertility (Conley and Hinshelwood, 2001).

Letrozole is widely used as a therapeutic alternative to treatments for reproductive disorders, including neoplasms (Ortega et al., 2013; Verma and Krishna, 2017). Comparative studies using several aromatase inhibitors (Lønning et al., 2003; Bhatnagar, 2007) have shown that letrozole is the most potent and selective drug that prevents intracellular estrogen synthesis and reduces hormone levels (Bhatnagar, 2007).

Letrozole is therapeutically effective in reversing reproductive illnesses related to infertility caused by hormonal imbalance, such as hypogonadism and polycystic ovary syndrome (Arroyo et al., 2019; Rambhatla et al., 2016). However, the effects of this anti-estrogen activity on gonadal activity over long periods are not clear. Reduction in estrogen levels can lead to a peripheral anabolic effect (Ortega et al., 2013), even in gonads, which causes morphological changes (Verma and Krishna, 2017; Lima et al., 2014). The effects associated with these treatments include changes in the expression of different receptors and proteins, as well as a reduction in the expression of aromatase and estradiol levels (Kondarewicz et al., 2011; Pilutin et al., 2014).

The blocking of aromatization in the prenatal androgen stage is important in establishing the pattern of sexual preference, sexual behavior, and male arousal (Olvera-Hernández et al., 2015). Even so, the effects of continued use of letrozole on the development of fertility and reproduction are not yet understood.

The aim of this study was to evaluate the effects of prolonged use of letrozole on the postnatal development of biometric reproductive parameters of rodents (Spix's yellow-toothed cavy; Galea spixii). This rodent species has been used as an experimental model for other rodents and humans to identify steroidogenic events related to reproduction and preservation (Santos et al., 2012, 2016, 2017a, 2021; Praxedes et al., 2017; Miglino et al., 2008).

## Material and methods

### Animals

The animals were collected from the Centro de Multiplicação de Animais Silvestres” (IBAMA 1478912/2011) of the Universidade Federal Rural do Semiárido, located in Mossoró, Brazil. The research was authorized by the Ethics Committee of the School of Veterinary Medicine and Animal Science of the University of São Paulo, São Paulo, Brazil (protocol number: CEUA FMVZ/USP nº 7781180516/2013) and by the Biodiversity Information and Authorization System (SISBIO nº 41910-4).

G. spixii of each sex (24 males and 24 females) were equally divided into treated and control groups. Age was established according to the phase of sexual development previously defined in previous studies as immature (15 days-of-age), pre-pubertal (30 days), pubertal (45 days), post-pubertal (90 days), and mature sexual (120 days) (Santos et al., 2012, 2014, 2017b).

The animals were bred in enclosures measuring 5 × 5 m2 and were paired in boxes according to sex. Each box (2.5 m2) had a sand floor, screen, and was covered with ceramic tiles. After delivery, the birth dates and gender identification of the puppies were recorded. The females and their puppies were separated and placed in individual enclosures (2.5 m2) to start the experiment. The diet available ad libitum included commercial rabbit feed, corn, local fruits, and water.

The experiment was conducted from June to December of 2016. According to the Laboratório de Metereologia e Climatologia at Universidade Federal Rural do Semiárido the average temperature during this time was 35.47°C, the relative humidity was 55.61%, rainfall amount was 5.70 mm, and average wind speed was 3 m/s. The duration of the days was approximately 12 h, with an average variation of 25 min.

### Administration of drugs and euthanasia

The first dose of letrozole (4,4’-(1H-1,2,4-triazol-1-metileno-dibenzonitrile, Femara®; Novartis Pharma Stein AG Stein, Switzerland) was at 15 days. Dosing continued weekly for 30, 45, 90, and 120 days. Each letrozole tablet (2.5 mg) was macerated and homogenized in 0.5 ml distilled water to produce a dose of 0.01 mg/kg body weight. The dose was delivered orally to each animal via a syringe.

All animals were anesthetized with 0.3 ml ketamine (Cristália Prod. Quím. Farm. Ltda, Brazil) and 0.3 ml xilasin (Syntec Ltda, Brazil). They were then euthanized with 0.4 ml of intrathoracic potassium chloride. The animals were weighed and the right testis and right ovary were collected, weighed, fixed in 4% paraformaldehyde (Sigma Chemical Co., USA) for 24 h, and sectioned.

### Weighing

Weight was used to indirectly assess the peripheral and/or tissue effects of letrozole's long- term action. Each rodent was weighed immediately prior to euthanasia using an WT21- LCD balance (Weightech, Davie, FL, USA). The right and right ovaries were weighed during collection using a M214A analytical balance (BEL Engineering, Monza, Italy).

### Morphological analysis

Sections of the testes and ovaries were fixed in 4% paraformaldehyde (Sigma-Aldrich, St. Louis, MO, USA) for 24 h, dehydrated in dilutions (70%–100%) of ethanol (Sigma-Aldrich), and embedded in paraffin. Sections of 5 μm were cut and mounted. Sections were stained using hematoxylin and eosin (Sigma-Aldrich) to investigate testicular and ovarian morphology. Following deparaffinization in xylol (Sigma-Aldrich, Wicklow, Ireland) at room temperature (RT) using two solutions for 10 min each, rehydration in a descending series of ethanol concentrations was performed (100%, 2 × 5 min; 5 min each for 90%, 80%, and 70%), followed by distilled water (RT, 5 min). The sections were then stained with hematoxylin for 30 s at RT, washed in running water for 10 min, stained with eosin at RT for 15 s, and washed in running water for 10 min. The slides were then dehydrated in increasing dilutions of ethanol (RT, 5 min 70%, 5 min 80%, 5 min 90%, 2 × 5 min 100%), cleaned in xylene (Sigma-Aldrich) at RT, 2 × 10 min, and mounted on Permount® (SP15-500; Thermo Fisher Scientific, Waltham, MA, USA). The analyses were performed by light microscopy.

### Statistical analyses

Body, testicular, and ovarian weights of 30-day-old animals were compared with the weight in 45, 90 and 120 day-old animals within and between the letrozole-treated and control groups using Shapiro Wilk and Bartlett test for normality and homoscedasticity (R Core Team, 2012), ANOVA followed by the Bonferroni test (IBM SPSS Statistics® software). Statistical significance was set at p <0.05.

## Results

### Body weight

**Figure 2:**
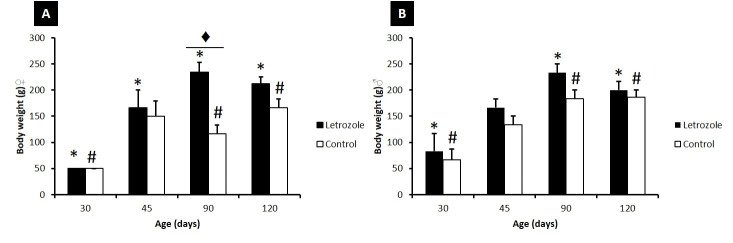
Letrozole effect on average body weight gain of female (A) and male (B) Spix's yellow-toothed cavy during sexual development in the letrozole and control groups. */# p<0.05 30 days vs. 45, 90 and 120 days; ♦ p<0.05 letrozole vs. control.

Body weight gain was greater in the group that received letrozole in both sexes and at all ages (Figure 2). Body weight in males in the control and treated groups increased up to 90 days, with a slight decrease evident at 120 days. The differences were statistically significant (p<0.05). Body weights in females also increased in both groups (treated and control) and were statistically significant (p<0.05).

### Ovarian weight

**Figure 3:**
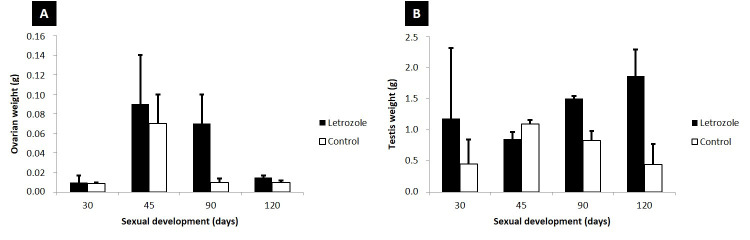
Effect of letrozole effect on mean testis and ovary weight gain during sexual development in the letrozole and control groups.

Ovarian weight gain was greater in the group that received letrozole at 30, 45, 90, and 120 days (Figure 3A). However, there was no significant difference between the age and experimental groups (p<0.05).

### Testes weight

Testicular weight gain was greater in the letrozole group, except at 45 days-of-age (Figure 3B). There was no significant difference between the age and experimental groups (p<0.05).

### Gonad histology

**Figure 4:**
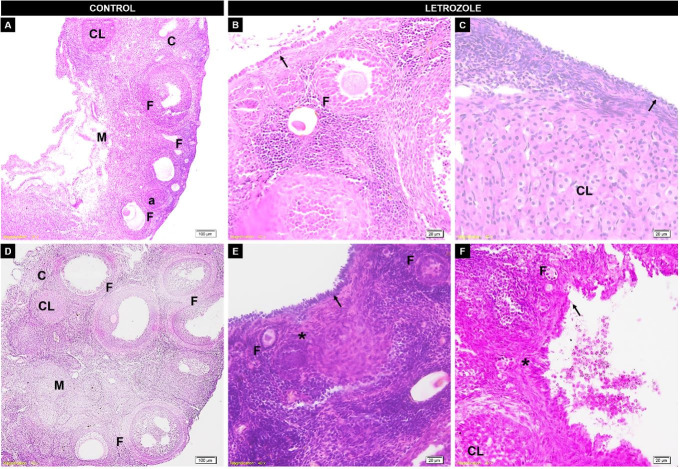
Effect of letrozole effect on the morphological development of the ovary of Spix's yellow-toothed cavy in the letrozole group (B-30 days, C-45 days, E-90 days, F-120 days) and the control group (A-30 days, D-90 days) groups. The arrow denotes the stratification of the epithelium. (*): Cellular disorder; (CL): corpus luteum; (C): cortical zone; (M): medullar zone; (F): follicles; (a): atresic follicle. A/C – The scale bar in panels A to C denote 100 μm (10× magnification). The scale bars in panels B, C, E, and F denote 20 μm (40× magnification).

In females not treated with letrozole, the surface of ovaries comprised simple cubic epithelium. The ovary was divided into two regions: a cortical zone with ovarian follicles at different stages of development, and a medullary zone with stroma and vessels. Letrozole altered the morphological development of the ovary (Figure 4). The continued use of letrozole led to stratification of the ovarian epithelium. At 120 days-of-age, micropapillary formation and cellular disorder were observed in the tunica albuginea. The treated groups did not show decreased follicular development.

**Figure 5:**
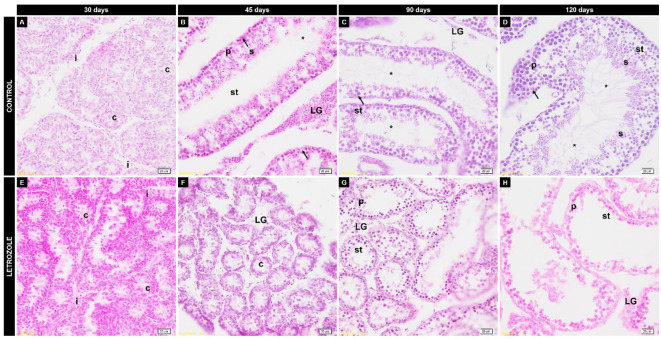
Effect of letrozole effect on the morphological development of the testis of Spix’s yellow-toothed cavy in the control (A-D) and letrozole (E-H) groups. The findings are evidence of spermatogenic regression. (c) testicular cords, (i) internal cord with interstitial cells, (st) seminiferous tubules with spermatogonia, (sc) Sertoli cells, (p) primary spermatocytes, (s) spermatids, (*) spermatozoa, and (LG) Leydig cells. Scale bars denote 20 μm (magnification 40×).

The testicular parenchyma of 30-day-old seminiferous tubules underwent a luminal process, with cells at different stages of the seminiferous epithelial cycle. At 45 days-of-age, the seminiferous tubules and interstitial tissue were well defined. The seminiferous tubules had a lumen, with a germinative epithelium being formed at different stages of the seminiferous epithelial cycle. In 90-day-old animals, seminiferous tubules contained lumina, seminiferous epithelial cells at different stages, and spermatozoa. At 120 days-of-age, the seminiferous tubules displayed luminal areas with cells at different stages of division, allowing their classification into stages of cellular associations as described previously (Curtis and Amann, 1981). However, the animals treated with letrozole showed a different morphology after 45 days-of-age. Regression of the morphological development of seminiferous tubes was observed (Figure 5). This finding implied a reduction in spermatogenesis and the absence of spermatozoa.

## Discussion

Prolonged use of letrozole increased body weight gain and gonads, induced infertility in males, and caused dysplasia in females. Letrozole causes an imbalance in the sex hormone steroid ratio, because it induces the reduction of estrogen levels by blocking the action of cytochrome P450 aromatase (Bhatnagar, 2007). This decrease in estrogen causes alterations in secondary and reproductive characteristics in both sexes (Rambhatla et al., 2016).

Our findings suggest that the likely increase in androgen levels at both the tissue and peripheral levels may result in an anabolic effect caused by the action of letrozole (Mareck et al., 2005; Ortega et al., 2013), which promotes greater gains in body weight and gonad weight in treated animals. The findings of the present study are supported by similar results in other studies, in which increased weight gain of letrozole-treated male rats was evident (Eshet et al., 2004), as well as in females (Pouliot et al., 2013) and of ovaries (Ortega et al., 2013).

Similarly, the use of the estrogenic inhibitor fulvestrant resulted in heavier male rats, similar to the testes (Oliveira et al., 2001). In contrast, letrozole caused weight gain in male rats at doses below 0.003 mg/kg (Pouliot et al., 2013). Furthermore, we demonstrated that prolonged use of letrozole modified the development of spermatogenesis, causing azoospermia. In rats, reducing estrogen levels in spermatogenesis resulted in germ cell hanging, giant cell, and vacuum cell formation in the seminiferous epithelium, in addition to degenerating cell clusters, membrane invagination, and lipofuscin accumulation in Leydig cells (Kondarewicz et al., 2011; Pilutin et al., 2014). Nevertheless, we observed that letrozole stratified the ovary lining epithelium, but did not alter oogenesis. We believe that prolonged use of letrozole may cause dysplasia in women. Our aim was to support the use of letrozole (Ortega et al., 2013) and tamoxifen (Lima et al., 2014), including proliferation and formation of epithelial cell multilayers, papillomatosis, increased nucleus size, and increased epithelial invagination.

The present study is clinically important, since letrozole is widely used as an alternative therapy for hormonal decompensation in both sexes (Arroyo et al., 2019; Ortega et al., 2013; Verma and Krishna, 2017). Long-term use of letrozole in Spix’s yellow-toothed cavy revealed that the inhibitor can be used for the development of secondary and reproductive traits in both males and females. This findings indicate the suitability of this rodent for studies of reproductive diseases that can affect men and women. The findings also support recommendations for further research on the steroidogenic effects of long-term use of letrozole.

## Conclusions

Letrozole affects the sexual development of Spix’s yellow-toothed cavy by promoting male infertility and inducing dysplasia in females upon prolonged use.
